# The sleeping crops of eastern North America: a new synthesis

**DOI:** 10.1098/rstb.2024.0192

**Published:** 2025-05-15

**Authors:** Natalie G. Mueller

**Affiliations:** ^1^ Department of Anthropology, Washington University, St Louis, MO 63130, USA

**Keywords:** domestication, North American, Eastern Agricultural Complex, landscape domestication, agrobiodiversity

## Abstract

Indigenous peoples in eastern North America domesticated a diverse group of annual crops. Several of these crops fell out of cultivation around the time of European colonization, and their domesticated forms are known only from the archaeological record. These crops have previously been characterized as *lost*, but in the context of a renaissance in Indigenous agriculture in this region, they are perhaps better understood as *sleeping*: this ancient agricultural system and its myriad ecosystem interactions could be reawakened. I briefly review the history of research on native eastern North American crops, and then synthesize recent research in terms of three themes: new models of domestication based on ecological, experimental and archaeological studies; new insights into the evolution of ancient agrobiodiversity; and an increasingly expansive understanding of the domesticated landscapes of ancient eastern North America. I conclude by suggesting some priorities for future research, and considering this sleeping agricultural system as a source of alternative crops and methods for the North American midcontinent in an era of rapid climate change.

This article is part of the theme issue ‘Unravelling domestication: multi-disciplinary perspectives on human and non-human relationships in the past, present and future’.

## 1. Introduction

**Figure 1.**
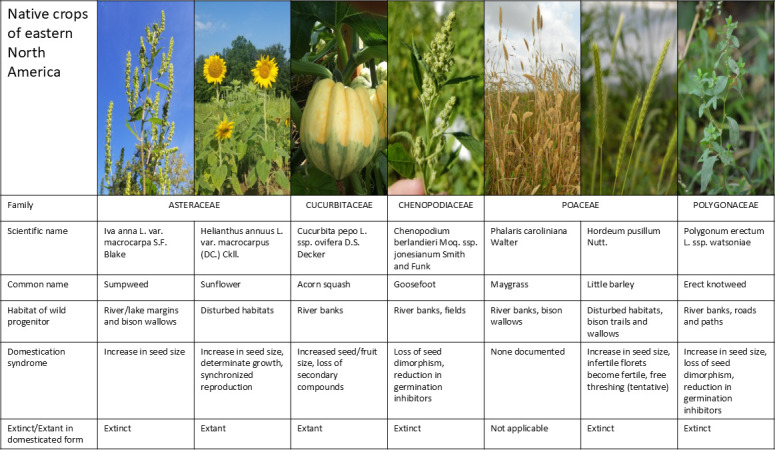
An overview of the native crops of eastern North America, sometimes referred to as the Eastern Agricultural Complex (EAC). See figure 2 for the locations and dates of earliest evidence for domestication of each species.

Indigenous peoples in eastern North America domesticated a diverse group of annual crops (figure 1). Several of these crops fell out of cultivation around the time of European colonization, and their domesticated forms are known only from the archaeological record. These have sometimes been characterized as ‘lost’ crops, as shorthand [1]. After hundreds of years of colonization and industrialization of eastern North America, during which the majority of Indigenous people were forced to leave their homelands, Indigenous food sovereignty movements are expanding the knowledge and practice of native North American agriculture once more [2]. In light of this renaissance, the ancient agricultural systems and crops of this region may be better characterized as *sleeping*, rather than lost: with the right combination of will, knowledge, access to land and resources and extant biodiversity, this system and its myriad ecosystem interactions could be reawakened.

Since the last synthesis of research on the domestication of eastern North American crops was published [3], archaeobotanical, experimental and ecological studies have generated novel hypotheses about how and why they were domesticated, the subsequent evolution of agrobiodiversity in this region, and the wider landscapes sculpted by Indigenous care. A synthetic view of the Eastern Agricultural Complex (EAC) reveals that it was uniquely well-suited to a landscape of highly productive but unpredictable floodplains, and that it relied on Indigenous care of mosaic landscapes, including tallgrass prairies, wetlands and forests. With climate change predicted to increase flood frequency and intensity in the midwestern United States, elements of this crop system could provide an alternative to current models of floodplain agriculture based on water control and energy-intensive monocropping of flood-sensitive crops. Reconceptualizing forests, prairies and wetlands as ecosystems that can provide food for humans could help make more space for them in the highly productive agricultural lands of eastern North America.

## 2. Background

**Figure 2.**
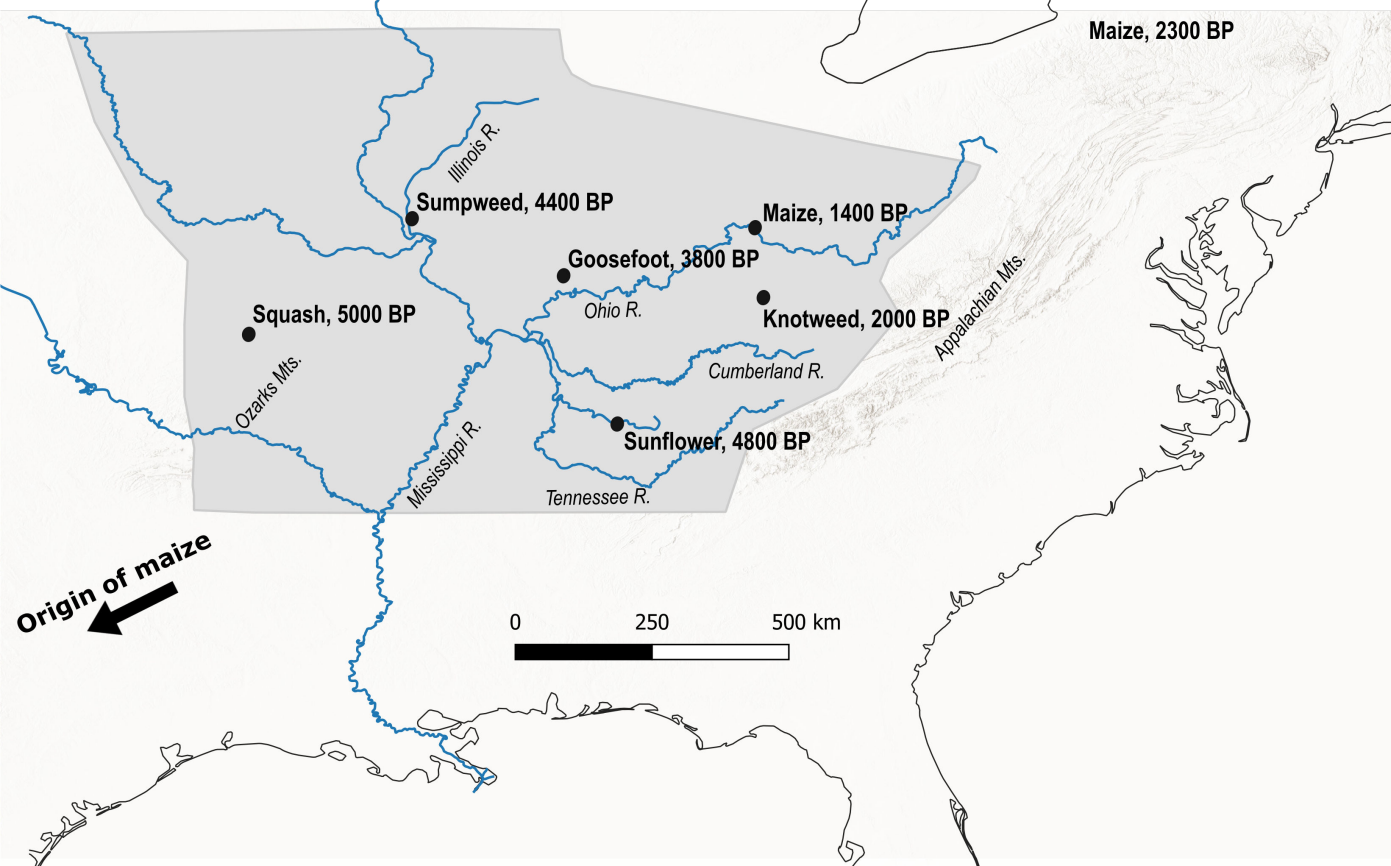
Map of the core area of the Eastern Agricultural Complex, shaded in grey. Locations and calibrated ages for the earliest domesticated assemblages are shown [3,9–12]. The earliest directly dated maize remains in the core area are also shown [13]. Maize was domesticated in Mexico and spread to eastern North America via the southwest, yet its earliest archaeological remains are from the northeast [14]. See text for details and figure 1 for an overview of each species.

Beginning in the 1920s−1930s with studies of large, desiccated plant assemblages from rockshelters [4,5], the study of ancient eastern North American agriculture intensified with the introduction of flotation as a standard archaeological procedure in the 1960s [6,7]. Flotation is an archaeological method for the recovery of plant tissue from sediment. Archaeological sediments are ‘floated’ in water, which allows lighter, organic inclusions to be separated by density. This methodological innovation allowed archaeologists to recover plant material from nearly every site, rather than only from sites with extraordinary preservation. Unlike in many other regions of the world, flotation was adopted as a standard procedure by both academic and many contract archaeologists, and has been applied to thousands of sites in the intervening decades [8]. As a result, the ‘core area’ of the EAC (figure 2) has one of the richest and most comprehensive paleoethnobotanical records in the world.

Aided by the invention of radiocarbon dating, pioneering botanists and their archaeologist collaborators began to argue for an ‘eastern agricultural complex’ composed of plants that are native to eastern North America, which predated maize-based agriculture by millennia (figure 1) [4,15]. By the end of the 1970s, it was clear that several species of native annual, small-seeded plants were abundant and ubiquitous in archaeological contexts across much of eastern North America. Botanists had identified these species, and early radiocarbon dating had revealed that they were cultivated beginning *ca* 7000 cal BP [9,16–20]. While many North American archaeologists were at first sceptical that these plants had been crops, the discovery of their seeds as common inclusions in human paleofaeces [21] and in clear storage contexts [5,22] were key pieces of evidence that led to their acceptance as seed crops that were once cultivated, but had fallen out of use by the historic era.

Beginning with Yarnell’s studies of sumpweed in the 1970s [19], scholars increasingly turned their attention to the question of whether or not the ‘lost’ crops were domesticated, and, if so, how, where and when their domestication occurred. Systematic studies revealed the evolution of distinct domesticated varieties of sumpweed (*Iva annua* L. var. *macrocarpa* S.F. Blake), goosefoot (*Chenopodium berlandieri* Moq.) [22–26] and little barley (*Hordeum pusillum* Nutt.) [27]. The histories of extant native crops also came into focus with the recovery of early squash (*Cucurbita pepo* L. ssp. *ovifera* D.S. Decker) [10], and sunflower (*Helianthus annuus* var. *macrocarpus* (DC.) Ckll.) remains [11]. A lively debate ensued about whether sunflowers and squashes were domesticated in eastern North America and/or Mexico, which has been settled in favour of eastern North America for both crops [28–32].

This generation of scholars of the EAC also developed the floodplain theory of domestication [7,33]. According to this theory, hunter-gatherers of the early and mid-Holocene first encountered the wild progenitors of crops like goosefoot and sumpweed on seasonally scoured river banks. These river banks were perpetual early succession environments, similar to a tilled field. This allowed dense stands of annual plants to form, which were easily harvested by people. The earliest domesticated assemblages of both goosefoot and sumpweed come from floodplain or floodplain-adjacent sites [3]. By *ca* 3000 cal BP, people were increasingly cultivating these floodplain plants in uplands. At the same time, there is clear paleoecological evidence for forest clearance and the expansion of early successional environments in uplands. Scholars of domestication wove these strands together to argue that farmers took floodplain-adapted plants out of their natural habitat, and that cultivation in uplands facilitated or accelerated the domestication process [34,35].

Other researchers asked when, where and how maize and other tropical crops arrived in eastern North America and were integrated into the existing crop system. This is an important question to resolve since maize is the focus of many Indigenous food sovereignty movements today, and was the most important crop cultivated by Indigenous people at the time of Euroamerican colonization. Maize is a cultural keystone species [36] for many Indigenous communities from eastern North America, as evidenced by religious practices, iconography and traditional histories, as well as farming and culinary traditions. The earliest evidence for maize cultivation in eastern North America, somewhat paradoxically, comes from the northeast and dates the arrival of this crop to *ca* 2300 cal BP (figure 2) [14,37]. How maize arrived in the northeast without leaving material traces of similar age in the core area, which has an exceptionally rich paleoethnobotanical record, remains mysterious. Reanalysis of purported early maize remains from the core area has repeatedly revealed that many specimens were either mis-identified or were not as old as associated dates [38–41]. However, recent direct dating of maize from the Middle Ohio Valley places the origins of maize agriculture there at *ca* 1400 cal BP (figure 2) [13]. Thus, the archaeological record as it currently stands suggests that maize largely bypassed the core area of the EAC, where agriculture had been practiced with native crops for centuries, and was first adopted by hunter-gatherers in the northeast by *ca* 2000 years ago. It then spread back down the Ohio River to the core area, where it was integrated into existing food production systems over the course of several hundred years. After *ca* 1100 cal BP, all lines of evidence are in agreement that maize quicky became an important crop across most of eastern North America [38,42]. Mexican squash domesticates (*C. pepo* L. ssp. *pepo* and *C. argyrosperma* C. Huber) were likely adopted around the same time, though it is usually hard to tell these apart from native squash domesticates in the archaeological record. Beans were adopted somewhat later, by *ca* 900 cal BP [43].

What all of these studies have in common is that they are focused on the evolution or adoption of annual seed crops. There are good reasons for this focus: (i) seed crops in general tend to preserve well in the archaeological record; (ii) they are particularly abundant in eastern North American assemblages, and (iii) their evolution during domestication caused changes to the plant parts that are preserved in archaeobotanical assemblages (the seeds)—not to mention the fact that the global food supply is increasingly dominated by annual seed crops [44]. However, this focus on annual seed crops would probably have seemed strange to the ancient Indigenous people of eastern North America, who relied on diverse animal and plant foods, including annual seed crops, nuts, fruits and roots, most of which came from managed perennial plants, not fields of annual crops [45,46]. This is particularly true of the Archaic peoples (*ca* 7000−3000 cal BP) whose actions were responsible for the domestication of several native seed crops.

At the end of the Late Archaic period, *ca* 3000 cal BP, the paleoecological record of eastern North America suggests that Indigenous people began to manage forests using fire [47–49]. At the same time, a period of severe flooding may have contributed to movement of people out of the major floodplains and to widespread social changes associated with the beginning of the Woodland period, including breakdowns in long distance exchange, cessation in monumental construction, and evidence for increasing sedentism in the form of material traces of permanent houses and storage of surplus food [50]. Subsequently, the EAC becomes increasingly visible and consistent in its component species through the Middle and Late Woodland periods (*ca* 2000−1100 cal BP)—a process that was perhaps facilitated by a new era of long distance exchange referred to as the Hopewell phenomenon [12,51]. Throughout this period, the remains of nuts, fruits and other edible plants remain abundant in the archaeological record, and there is no reason to suppose that access to forests, prairies or wetlands was limited by the expansion of cultivation or increasing sedentism. Finally, during the Mississippian period (*ca* 1100−500 cal BP), farmers integrated maize and other new crops from Mexico into existing agricultural systems. Maize gradually became more important, as villages grew into towns or even cities, in the case of Cahokia. Even at Cahokia, the largest Mississippian settlement, the diversity of the food system and access to multiple kinds of ecosystems is apparent, and some farmers in the midcontinent continued to grow the older native crops right up until Euroamerican colonization began [52,53].

Here, I synthesize recent EAC research and relate it to this rich literature in terms of three themes:

(1) new models of domestication that have emerged from experimental and ecological research with crop progenitor species;
(2) new insights into the agrobiodiversity of the EAC;
(3) the application of the idea of landscape domestication to this region, which allows us to shift our focus from documenting domestication in annual species to documenting changes in ecosystems and their distributions.

I characterize the EAC as highly diverse and responsive (plastic) at both macro and micro scales. I close by suggesting some priorities for future research, and considering this sleeping agricultural system as a source of alternative crops and methods for the North American midcontinent in an era of rapid climate change.

### 3. New models of domestication

The floodplain theory of domestication outlined above was proposed mainly on the basis of observations of free-living populations of goosefoot and sumpweed [33]. These species are often found in dense stands in open, seasonally inundated habitats along rivers that were recognized by many theorists of domestication as frequently disturbed habitats similar to cultivated fields [54,55]. But there is a key difference between floodplains and fields: predictability. With a series of studies of erect knotweed, another floodplain-adapted crop progenitor, the floodplain theory has been elaborated to include an understanding of bet-hedging and developmental plasticity as strategies used by plants that have evolved in unpredictable environments [35,56–58].

Unpredictable environments change sporadically, making it impossible for immobile plants to optimize for any particular set of conditions. For example, one year a midwestern floodplain may provide a well-watered environment for germination and early growth that slowly dries out throughout the summer and autumn. In this scenario, a seed that germinates in early spring and grows rapidly will be successful at reproducing itself. In another year (and increasingly frequently), the floodplain may be inundated in the middle of the summer, killing all of the individuals that germinated in the spring before they are able to reproduce. In this year, the population will go extinct unless it also has recalcitrant seeds ‘waiting’ in the seed bank that can germinate the following spring. Because of this unpredictability, many annual plants adapted to floodplains, including goosefoot [59] and erect knotweed [35], have evolved germination heteromorphism: they produce two or more types of seeds, with different requirements for germination [60,61]. Likewise, developmental plasticity is greater in plants that are ‘weedy’, in the ecological sense [62] of adapted to unpredictable disturbance, than in their non-weedy relatives [63]. This allows weedy plants to change aspects of their phenotype in response to shifting environmental conditions during their lifetimes.

After observing free-living populations of erect knotweed for several years, growing plants under experimental conditions and studying changes in seed phenotypes over time in the archaeological record, I argued that both bet-hedging and plasticity were reduced or eliminated during domestication as a result of three specific human practices [58]:

(1) The archaeological and paleoecological records suggest that from approximately 3000 years ago, people increasingly removed floodplain-adapted plants from the unpredictable environments in which they evolved and created stable environments for them in clearings above the floodplain.
(2) There is also clear archaeological evidence for seed storage, some of which seems to have been ‘seed stock’ of superior quality, intended for planting the following year.
(3) In addition, experimental evidence suggests that plants grown at densities lower than that of typical free-living stands are more productive, which would have encouraged people to thin out fields or managed stands.

All three of these practices would favour seeds with minimal germination inhibitors that will sprout quickly when planted, while removing or reducing the selective pressures that maintained seed heteromorphism and dormancy in the wild. The result is the evolution of larger seeds with minimal seed protections and loss of the bet-hedging type in the archaeological record for both goosefoot and knotweed.

Our experiments also revealed that one of the most important phenotypes used to study the evolution of plant domestication—the reduction of germination inhibitors—is highly plastic in some crop progenitors and may have changed first as a developmental response to new growth environments created by cultivation, before any evolutionary change occurred. By growing erect knotweed and goosefoot in gardens, my colleagues and I have been able to produce harvests that are more similar to ancient domesticated assemblages than they are to harvests produced by their free-living parent populations [59]. This seems to be at least partly a response to greater light availability in gardens that are tended by weeding and maintaining low crop density [58]. These may be cases of adaptive transgenerational plasticity, in which a mother plant prepares her offspring to take advantage of the open environment she senses by producing seeds that germinate more easily [64]. We have described the domestication of erect knotweed and goosefoot as taking place via genetic assimilation: a trait that was once plastic (seeds with different germination requirements in proportions determined by environmental factors) becomes fixed (only seeds with minimal germination requirements are produced). Theoretically, this should occur when there is a fitness cost to maintaining plasticity [65]. In this case, seeds that germinate later and have slower early growth would be at a disadvantage in an environment where humans routinely remove the smallest plants when thinning out stands, and would no longer be necessary to hedge against adverse disturbances [35]. Our results echo experimental research with teosinte, the wild progenitor of maize, which showed that teosinte plants are also capable of spontaneously producing maize phenotypes [66,67]. Likewise, experiments comparing the plasticity of crop progenitors to their descendent crops have repeatedly shown that plasticity is reduced during domestication [68,69]. These results suggest that genetic assimilation may have played a role in the domestication of many crops.

We have also gained new insight into the domestication of the EAC crops by taking a comparative perspective on the evolution of annual, small-seeded crops. Many small-seeded crops, including several of the EAC crop progenitors, were previously dispersed in the dung of grazing animals (via endozoochory). Such plants benefit from directed dispersal to open, nitrogen-enriched habitats, but also need to produce small seeds with robust protections in order to survive digestion. By taking over as seed dispersers, humans relaxed the selective pressures that maintain these phenotypes [70]. We conducted a study of crop progenitors in a remnant tallgrass prairie where bison have been reintroduced, and found that sumpweed, little barley and maygrass were all dispersed in bison dung. Moreover, we found that bison grazing and wallowing creates habitat for annual plants in tallgrass prairies. These habitats were dominated by dense stands of EAC crop progenitors growing in association—something that had not been previously observed elsewhere. We argued that, in addition to floodplains, ancient hunter–gatherers would have encountered EAC crop progenitors as they travelled through tallgrass prairies along bison trails, and that this could have been another locus of domestication: a place where people began to harvest and tend stands of crop progenitors [71].

These new insights into the domestication process in eastern North America suggest three general conclusions:

(1) Relaxation of selective pressures that were previously very strong—such as passing through the digestive tract of a bison or experiencing a mid-summer flood—was just as important as novel selective pressures imposed by humans in shaping domesticated plants.
(2) Reduction of plasticity in response to a more stable environment maintained by humans may be a common factor in the domestication of many weedy plants.
(3) Ecological relationships between plants and non-human animals should not be ignored in explaining domestication, even in places where no animals were domesticated (such as eastern North America).

### 4. Agrobiodiversity of the Eastern Agricultural Complex

Initial domestication is continuous with subsequent on-farm crop improvement and diversification in terms of both cultural and biological processes [72]. Throughout the Holocene, heterogeneity in human agricultural practices and preferences, as well as non-uniform systems of germplasm exchange, created and maintained an enormous amount of agrobiodiversity, even on a local scale. Increasingly, the archaeological record suggests that the supposed ‘domestication bottleneck’, in which diversity in crop gene pools was assumed to have steeply dropped in comparison to the wild parent population during initial domestication, may actually only have occurred recently with colonialism and industrialization [73,74]. These global insights into the evolution of agrobiodiversity are also reflected in several specific cases from eastern North America. A study of ancient sunflower genomes revealed that multiple distinct landraces were grown by the same Ozarks communities, and that much of this diversity is absent from the modern sunflower gene pools [75]. Morphological studies of erect knotweed [76], goosefoot [3,23,77,78] and sumpweed [79] show nonlinear change in phenotypes over time and spatial heterogeneity in domesticated types. For erect knotweed and goosefoot, specific ancient landraces have been described, which consistently differ in terms of phenotype. In the case of goosefoot, different landraces were grown by the same communities, presumably for different uses or in slightly different environments [59]. In the case of erect knotweed, landraces map onto different communities of practice. Communities in the Ozarks, eastern Missouri, and western Illinois maintained distinct varieties during a time of great social change (the aggregation and abandonment of the urban centre at Cahokia) [76].

The agrobiodiversity of the EAC is even more striking when we zoom out from landrace diversity in seed crops to examine the food system as a whole. Eastern North American agriculture was never focused on a single staple crop. Even at the time of European colonization, when fewer crops were grown than in previous centuries, most communities grew multiple kinds of maize, beans, squash and sunflowers in polyculture, along with many other minor crops and managed perennial food plants. The peak of crop diversity in eastern North America was probably *ca* 1100−600 cal BP, when the older native crops were integrated with newly introduced maize, Mexican squashes and (eventually) beans, to create a crop system that included no fewer than ten annual plant species from six different families (figure 1). The diversity of this suite of annual crops cries out for investigations into the cropping system: where and when was each crop grown?; how did they work together or compete? and how did each one contribute to cuisine and food security?

Since many of these crops are sleeping, we are only beginning to answer these questions through a combination of inferences drawn from more recent Indigenous practices, experimentation and close examination of archaeological context. It is likely that crops would have been grown in polycultures and that farmers would have taken advantage of the hydrography of floodplains to cultivate crops that have different tolerances of flooding [52]. Polyculture experiments with certain sleeping crops suggest synergies: erect knotweed and goosefoot are more productive when grown together than either is on its own [80]. This polyculture is functionally similar to maize and squash in the Three Sisters system: in both systems, one species serves as an edible ground cover without competing with the taller crop.

While the diversity of annual crops cultivated by Indigenous farmers in eastern North America is impressive, these were still only part of the overall food system. I will now turn to this larger food system, which can be described as a domesticated landscape.

### 5. Landscape domestication

A domesticated landscape is one in which humans have changed the composition and distribution of ecosystems in ways that make food and other resources more abundant, accessible or predictable [81]. Around the world, historical ecologists working with Indigenous and local experts have made profound discoveries about the ways in which seemingly pristine ecosystems were shaped by human knowledge and practice over the course of thousands of years. The approaches taken by historical ecologists in other regions are complicated in eastern North America by the scale and severity of colonial destruction: nearly all of the forests have been clear cut, prairies have been transformed into industrial row crop farms and the vast majority of rivers have been modified, transforming floodplains and shrinking wetlands. Eastern North American landscapes are dominated by simplified industrial ecosystems of commodity crops. Native species continue to be driven to extinction by introduced predators and pathogens and settler colonial land use. Most importantly, the vast majority of the Indigenous people of this region were forcibly removed from their homelands, interrupting their care of the land and making it harder for communities to practice and reproduce ecological knowledge.

**Figure 3.**
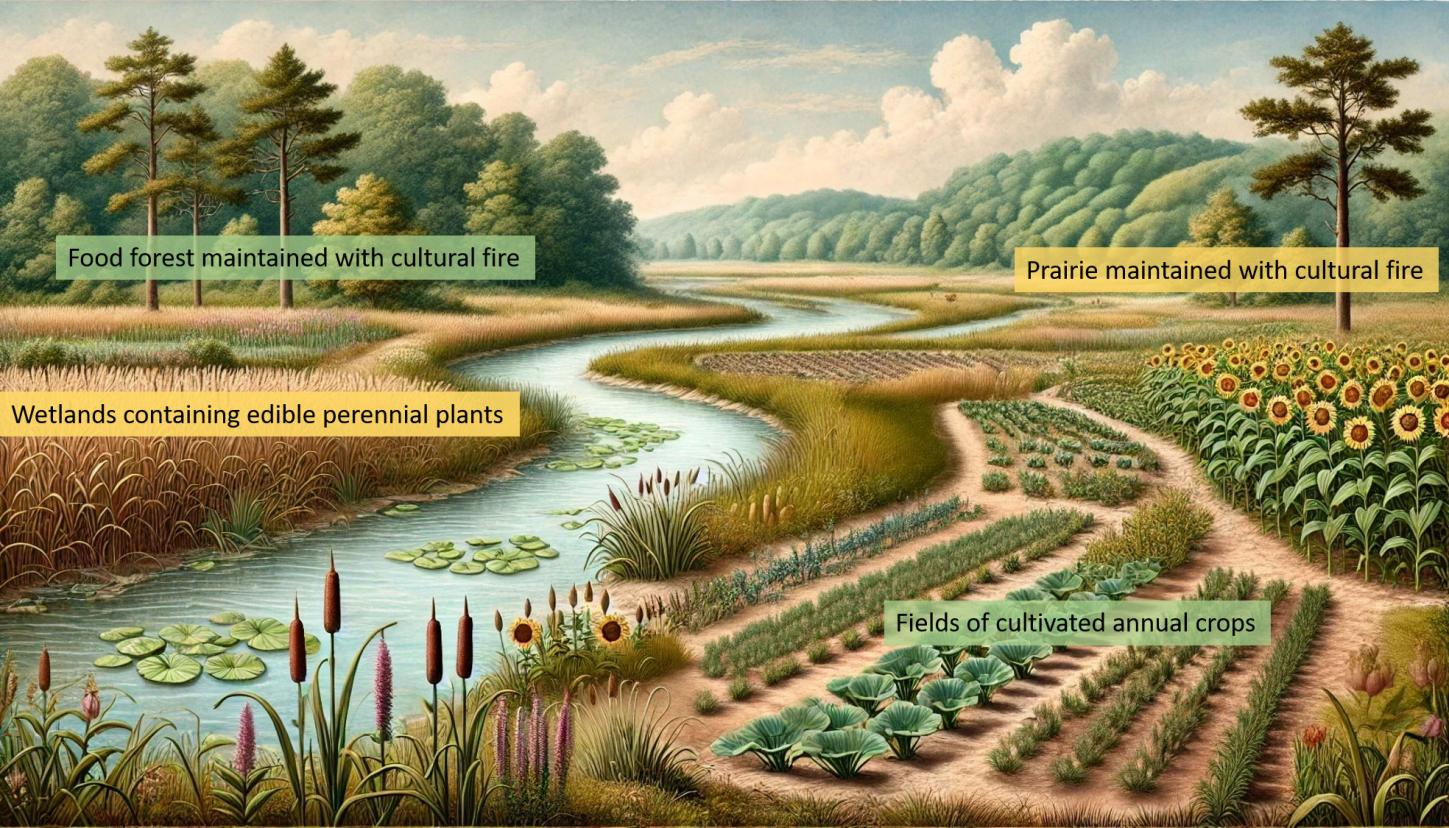
Generalized view of the domesticated landscape of eastern North America. This image was generated using ChatGPT 4 and DALL·E.

Nevertheless, the broad strokes of ancient domesticated landscapes in eastern North America have been outlined (figure 3) [82]:

(1) *Forests*. In a series of studies comparing historical maps of tree distribution to modern forests, ecologists have convincingly argued that Indigenous people maintained pyrophytic forests full of nut-bearing trees using controlled fire for thousands of years [83,84]. This agrees with the paleoecological record, which shows a shift in prevalence of these same trees around 3000 years ago [47–49,85]. Cultural fire increased the prevalence of food-rich oak–hickory forests. The archaeological record attests to the importance of these species for human diets throughout the Holocene [86]. There is also tentative evidence for human dispersal of fruit trees, such as persimmons, to create managed groves [87]. This is a topic that is ripe for further investigation.
(2) *Prairies*. The tallgrass prairies of the Great Plains once extended into humid, mostly forested eastern North America in an anomalous swathe referred to by ecologists as the Prairie Peninsula. Scholars have noted that the historic prairies extended into a climate zone where they are not expected to exist without frequent fire, and have argued over whether or not they were anthropogenic for a century [88–91]. The latest paleoecological evidence supports a scenario in which cultural fire maintained the Prairie Peninsula after the onset of more humid conditions *ca* 5000 cal BP [92]. The prairies were (and are) important ecosystems for Indigenous people in this region because they are habitat for bison, a cultural keystone species for many tribes, but also because they contain edible and medicinal plants, including some of the EAC crops [93].
(3) *Wetlands and floodplains*. To date, there is no clear evidence that any eastern North American society engaged in hydroengineering for food production. Nowadays in eastern North America, such efforts are mostly aimed at excluding or controlling the flow of water (e.g. levees, canalization and dams). Considering the fact that ancient Indigenous societies were accomplished engineers who frequently built monumental earthen constructions, this absence of engineering in the floodplains seems like a conscious decision. All of the native crops of the EAC are floodplain adapted to one extent or another, though their exact tolerances to flooding at different moments in their development have not yet been investigated. This is also true of river cane (*Arundinaria gigantea*), a multiuse fibre plant of high cultural and economic importance across eastern North America. By growing plants that can tolerate flooding, Indigenous farmers could make use of the topographic variability of unmodified floodplains, with their natural levees, backwater lakes and perennial wetlands. This laissez-faire approach to farming in the floodplain also preserved the habitat of fish, shell fish and aquatic birds, which were important sources of food that have been greatly reduced by Euroamerican modification of floodplains for agriculture.

The only hint of Indigenous terraforming in the floodplains of eastern North American (other than ceremonial–political earthen mounds) are the anthropogenic wetlands in the urban core of Cahokia, a city that flourished in the vast floodplain at the confluence of the Mississippi and Missouri rivers from *ca* 1100−650 cal BP. In contrast to modern hydroengineering features designed to exclude water from cultivated lands, these ‘borrow pits’ (sediment sources for earthen monuments) invite water in, creating perennial or seasonal wetlands within the floodplain. There is also some paleoethnobotanical evidence for the harvesting of perennial wetland species, such as American lotus, a species that is still harvested for special purposes by some descendent communities [94]. The pollen of this species has also been recovered from the borrow pits at Cahokia, though it does not grow there today [95]. At least two of the sleeping crops of the EAC, little barley and sumpweed, are abundant in and around the borrow pits, which begs the question of whether and to what extent these anthropogenic wetlands were sites of urban food production.

Historical accounts by European explorers describe mosaic landscapes composed of cultivated fields, forests, prairies and wetlands in close spatial proximity (figure 3) [96,97]. This is one of the hallmarks of a domesticated landscape: disparate ecosystems and resources are brought into association through human management; edges become abundant [98,99]. This allows people to gather food and other resources close to their homes, to easily predict where and when resources will be available or to travel less than would otherwise be possible. The effects of this form of management are evident in the diversity and variability of the paleoethnobotanical record in this region. The seeds and fruits of ‘wild’ plants are always abundant, even as domestication occurs and crop seeds increase in ubiquity. But the evolutionary effects of Indigenous management on plants other than annual seed crops in this region are almost entirely unknown, and represent a frontier of domestication research.

### 6. Awakening sleeping seeds: plasticity and diversity as pillars of resilient food production

Future research on the EAC should harness the potential of one of the richest paleoethnobotanical records in the world, while integrating methods that are seldom used in this region. These include microbotanical analysis (pollen, phytoliths and starch), botanical survey and population genetics of crop wild relatives and their symbionts, ancient DNA, historical research and ethnographic and ethnobotanical research with descendent communities. These methods could shed light on less-visible components of the agricultural complex, especially perennial crops. Experimental and ecological methods can be used to understand the potentials and tolerances of the species that were part of ancient food systems: we do not know how useful these foods might be in the context of climate change because we do not know how they respond to stresses like flooding and drought. Given the richness of the existing paleoethnobotanical record and the availability of high-quality paleoclimatic studies, eastern North American can contribute to an understanding of how ancient people and ecosystems responded to climate change. In order to realize this potential, we will need to collect new, high temporal resolution paleoecological data and to digitize the existing paleoethnobotanical and zooarchaeological records, which are currently impossible to analyse as a whole.

Scholars of domestication and ancient agriculture have a responsibility to share the diversity and ingenuity we see in the archaeological record, especially with those who see the history of human ecology as a steady march of increasing ecological devastation culminating in our current crisis. The methods of ancient Indigenous farmers in eastern North America provide an alternative to the stark choice between maximizing food production and protecting biodiversity: they did both. There are various ways that the crops and techniques that were part of this system could be integrated into contemporary food production. For example, in eastern North America, it is increasingly difficult to protect floodplain fields from floods of increasing severity that occur less predictably [100]. Cultivating wetland or floodplain-adapted crops instead of industrial maize and soy could be a low-input way out of this trap. In some less productive parts of eastern North America, farmlands are becoming re-forested [101]. These could be managed using fire as food forests, increasing their value to both human and non-human animal communities. Prairies are already making a comeback as sites of cultural and ecological restoration and food production as bison herds proliferate, especially on Tribal lands [102].

It is impossible to fully recreate the ancient domesticated landscapes of eastern North America, but emulating aspects of them could result in a more diverse food production system with a landscape that provides (in twenty-first century environmental management terms) ‘ecosystems services’, including maintenance of biodiversity, topsoil retention, carbon sequestration, water filtration and more. But what about justice for the descendants of the people who cared for these lands in the past? In other regions, historical ecologists have worked closely with local and Indigenous communities to understand the landscapes they study—in some cases, with explicitly activist agendas of restoring access to or control over ancestral homelands. This has generally not been true in eastern North America. If historical ecologists working in eastern North America consciously emulate this approach in the future, we would need to start with a different set of questions: What are the goals of descendent communities, both for the landscapes they manage and for those they have lost access to? What are legal or economic means of returning land and plant genetic resources to Indigenous management in eastern North America, in the context of historical erasure and removal? Can scientific research about domestication and ancient agriculture contribute to the goals of Indigenous food sovereignty movements? This approach would probably result in research projects with applied and/or actionable results. For example, the Rivercane Restoration Alliance is a collaboration between the US Army Corps of Engineers, the Nature Conservancy and Tribal Nations to conduct multidisciplinary research and facilitate restoration projects that increase access to this cultural keystone species. My own lab has taken small steps in this direction by maintaining a seed bank of sleeping crop progenitors, which to date has distributed seed to 25 educational institutions, Indigenous farmers and students. We also maintain a website with growing guides, which provide practical advice on how to cultivate and process each species. In the future, we hope to further refine our understanding of these species through experimentation, and contribute to reintegrating them into local and Indigenous food systems.

## Data Availability

This article has no additional data.
